# Probiotics: Prevention of Severe Pneumonia and Endotracheal Colonization Trial—PROSPECT: a pilot trial

**DOI:** 10.1186/s13063-016-1495-x

**Published:** 2016-08-02

**Authors:** Deborah J. Cook, Jennie Johnstone, John C. Marshall, Francois Lauzier, Lehana Thabane, Sangeeta Mehta, Peter M. Dodek, Lauralyn McIntyre, Joe Pagliarello, William Henderson, Robert W. Taylor, Rodrigo Cartin-Ceba, Eyal Golan, Margaret Herridge, Gordon Wood, Daniel Ovakim, Tim Karachi, Michael G. Surette, Dawn M. E. Bowdish, Daphnee Lamarche, Chris P. Verschoor, Erick H. Duan, Diane Heels-Ansdell, Yaseen Arabi, Maureen Meade, For the PROSPECT Investigators and the Canadian Critical Care Trials Group

**Affiliations:** 1 Department of Medicine, McMaster University, McMaster Health Sciences Center, Room 2C11, 1200 Main Street W, Hamilton, ON Canada; 2 Department of Clinical Epidemiology and Biostatistics, McMaster University, Hamilton, ON Canada; 3 Public Health Ontario, Toronto, ON Canada; 4 St. Joseph’s Health Center, Toronto, ON Canada; 5 Department of Medicine, University of Toronto, Toronto, ON Canada; 6 Department of Surgery, University of Toronto, Toronto, ON Canada; 7 Interdepartmental Division of Critical Care, University of Toronto, Toronto, ON Canada; 8 Research Center of the CHU de Québec – Population Health and Optimal Health Practices Research Unit, Université Laval, Quebec City, QC Canada; 9 Department of Medicine, Université Laval, Quebec City, QC Canada; 10 Department of Anesthesiology and Critical Care, Université Laval, Quebec City, QC Canada; 11 Division of Critical Care Medicine, St. Paul’s Hospital and University of British Columbia, Vancouver, BC Canada; 12 Center for Health Evaluation and Outcome Sciences, St. Paul’s Hospital and University of British Columbia, Vancouver, BC Canada; 13 Division of Critical Care, University of Ottawa, Ottawa, ON Canada; 14 Division of Critical Care Medicine, Vancouver General Hospital, Vancouver, BC Canada; 15 Department of Critical Care Medicine, Mercy Hospital, St Louis, MO USA; 16 Division of Pulmonary and Critical Care Medicine, Mayo Clinic, Rochester, MN USA; 17 Department of Anesthesia & Critical Care, Vancouver Island Health Authority, Victoria, BC Canada; 18 Department of Pathology and Molecular Medicine, McMaster University, Hamilton, ON Canada; 19 Biochemistry and Biomedical Sciences Department, McMaster University, Hamilton, ON Canada; 20 King Saud Bin Abdulaziz University for Health Sciences and King Abdullah International Medical Research Center, Riyadh, Saudi Arabia

**Keywords:** Critically ill, Infection, Intensive care, Probiotics

## Abstract

**Background:**

Probiotics are live microorganisms that may confer health benefits when ingested. Randomized trials suggest that probiotics significantly decrease the incidence of ventilator-associated pneumonia (VAP) and the overall incidence of infection in critically ill patients. However, these studies are small, largely single-center, and at risk of bias. The aim of the PROSPECT pilot trial was to determine the feasibility of conducting a larger trial of probiotics to prevent VAP in mechanically ventilated patients in the intensive care unit (ICU).

**Methods:**

In a randomized blinded trial, patients expected to be mechanically ventilated for ≥72 hours were allocated to receive either 1 × 10^10^ colony-forming units of *Lactobacillus rhamnosus* GG or placebo, twice daily. Patients were excluded if they were at increased risk of *L. rhamnosus* GG infection or had contraindications to enteral medication. Feasibility objectives were: (1) timely recruitment; (2) maximal protocol adherence; (3) minimal contamination; and (4) estimated VAP rate ≥10 %. We also measured other infections, diarrhea, ICU and hospital length of stay, and mortality.

**Results:**

Overall, in 14 centers in Canada and the USA, all feasibility goals were met: (1) 150 patients were randomized in 1 year; (2) protocol adherence was 97 %; (3) no patients received open-label probiotics; and (4) the VAP rate was 19 %. Other infections included: bloodstream infection (19.3 %), urinary tract infections (12.7 %), and skin and soft tissue infections (4.0 %). Diarrhea, defined as Bristol type 6 or 7 stools, occurred in 133 (88.7 %) of patients, the median length of stay in ICU was 12 days (quartile 1 to quartile 3, 7–18 days), and in hospital was 26 days (quartile 1 to quartile 3, 14–44 days); 23 patients (15.3 %) died in the ICU.

**Conclusions:**

The PROSPECT pilot trial supports the feasibility of a larger trial to investigate the effect of *L. rhamnosus* GG on VAP and other nosocomial infections in critically ill patients.

**Trial registration:**

Clinicaltrials.gov NCT01782755. Registered on 29 January 2013.

**Electronic supplementary material:**

The online version of this article (doi:10.1186/s13063-016-1495-x) contains supplementary material, which is available to authorized users.

## Background

Probiotics are live microorganisms that may have health benefits when ingested [1]. Probiotics have been studied in randomized controlled trials in a variety of conditions in the community and hospital setting [2–5]. In the intensive care unit (ICU), probiotics have been studied for the prevention of ventilator-associated pneumonia (VAP), potentially by enhancing intestinal barrier function and reducing the load of pathogenic bacteria [6, 7]. As the commonest nosocomial infection in the ICU, VAP is associated with a two-fold attributable risk of death, and an attributable cost of US$ 10,000–13,000 per patient [8].

A recent meta-analysis of 23 randomized controlled trials suggested that administering probiotics to critically ill mechanically ventilated patients is associated with a 25 % reduction in the incidence of VAP and an 18 % reduction in the incidence of all nosocomial infections [9]. A subsequent Cochrane review [10] of eight randomized controlled trials that enrolled 1083 patients in total and compared various single or combined probiotics to a control intervention (placebo, glutamine, fermentable fibre, peptide, chlorhexidine) suggested that probiotics might significantly decrease the incidence of VAP (odds ratio, 0.70; 95 % confidence interval, 0.52–0.95). One high-quality randomized controlled trial compared a combination of oropharyngeal plus gastric *Lactobacillus rhamnosus GG* with placebo in 146 patients who were expected to remain intubated for at least 72 hours [11], and found that those who received *L. rhamnosus GG* had lower rates of VAP (relative risk, 0.46; 95 % confidence interval, 0.26–0.82). The three randomized controlled trials that reported diarrhea as an outcome found a trend toward a lower incidence with probiotics (odds ratio, 0.72; 95 % confidence interval, 0.47–1.09; very low quality evidence); however *Clostridium difficile* was not analyzed. There were no reports of nosocomial infections caused by the probiotic organisms in these trials. Although promising, these small (*n* = 50–300), mostly single-center randomized controlled trials generate results that have uncertain internal and external validity. Therefore, while promising, current knowledge does not provide sufficient evidence to draw firm conclusions about the efficacy and safety of probiotics for the prevention of VAP in the ICU [10].

**Fig. 1:**
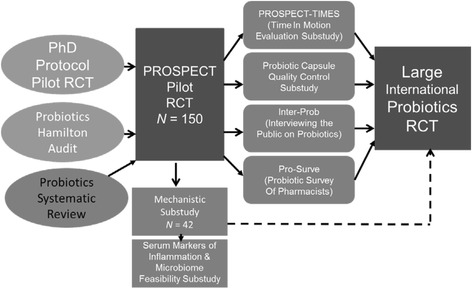
The probiotics in ICU research program. The PROSPECT pilot trial is part of the probiotics in ICU Research Program

In the context of a research program on probiotics in critical illness (Fig. 1), we conducted a pilot trial to evaluate the feasibility of a large randomized controlled trials in critically ill patients, investigating whether orally ingested *L. rhamnosus* GG prevents VAP and other nosocomial infections (Probiotics: Prevention of Severe Pneumonia and Endotracheal Colonization Pilot Trial, the PROSPECT pilot trial; NCT01782755). The criteria for feasibility were successful and timely pilot trial recruitment, high adherence to protocol, minimal open-label use of probiotics, and a VAP incidence of at least 10 %. We also report the results of three substudies nested within this pilot trial.

## Methods

### Study design

We conducted a randomized concealed blinded parallel group trial in 14 ICUs in Canada and the USA, in which patients were allocated to placebo or probiotic in a fixed allocation ratio of 1:1 using the randomize.net website. Randomization was stratified by ICU and by medical, surgical, or trauma status, in variable unspecified block sizes. Further details are reported in the protocol manuscript [12].

We included patients ≥18 years of age who were expected to be mechanically ventilated ≥72 hours. We excluded patients who: (a) had been mechanically ventilated for more than 72 hours at the time of screening, (b) were immunocompromised (HIV <200 CD4 cells/μl, chronic immunosuppressive medications, prior organ or hematological transplant, absolute neutrophil count <500 cells/μl), (c) had an increased risk of endovascular infection (history of rheumatic heart disease, congenital heart defect, mechanical valve, endocarditis, endovascular graft, or permanent endovascular device, such as hemodialysis catheter, pacemaker, or defibrillator), (d) had gastroesophageal or intestinal injury or recent surgery of the esophagus, stomach, bowel, hepatobiliary tree, spleen, or pancreas in the previous 72 hours, suspected or documented bowel ischemia, and severe acute pancreatitis (pancreatitis with any organ dysfunction), (e) had strict contraindication or inability to receive enteral medications, (f) were pregnant, (g) were undergoing withdrawal of life support, and (h) were enrolled in this or an ongoing related trial.

After written informed *a-priori* consent was obtained from the patient or the patient’s substitute decision maker, the local study pharmacist obtained the allocation from www.randomize.net.

### Trial interventions

Patients allocated to the intervention received 1 × 10^10^ colony-forming units of *L. rhamnosus* GG (Culturelle, Locin Industries, Ltd) in one capsule suspended in tap water, administered via gastric or duodenal tube twice daily while in the ICU. Patients allocated to placebo received microcrystalline cellulose suspended in tap water, identical in appearance and consistency to the probiotic, and administered similarly. Identical placebo ensured blinding of all possible parties (patient, family, clinicians, laboratory, and research personnel, including the biostatistician). The probiotic and placebo were prepared by the manufacturer of *L. rhamnosus* GG, Culturelle, as used in a recent trial [11]. Patients received the study product until discharge from ICU (administered in capsule form or as a suspension if no feeding tube was in place), on isolation of *Lactobacillus* species from a sterile site or reported as the sole or predominant organism in a culture from a non-sterile site, or 60 days after randomization.

All other management, including VAP prevention strategies, was at the discretion of the ICU team and according to local practice.

### Feasibility outcomes

The four feasibility outcomes were: (a) successful recruitment, defined as ≥2 patients per month on average per site (total 150 from all study sites) over 1 year, (b) adherence to protocol, defined as ≥90 % of the prescribed intervention being administered during the ICU stay, (c) minimal contamination, defined as ≤5 % of patients receiving open-label probiotic, in either group, during the ICU stay, and (d) a VAP incidence of ≥10 % as defined by the Centers for Disease Control and Prevention [13].

### Clinical outcome measures

Clinical outcome measures used in this pilot were those we will record in the main PROSPECT trial and included: the primary outcome measure, VAP; other ICU-acquired infections, defined using an adaptation of the International Sepsis Forum Consensus Conference on Definitions of Infections in the ICU [14]; diarrhea (defined as either ≥3 stools/day [15] or ≥1 Bristol type 6 or 7 stool, where type 6 is described as mushy stool, and type 7 is described as entirely liquid [16]), antibiotic-associated diarrhea (defined as ≥3 stools/day occurring within 24 hours of antibiotics) [17] and *C. difficile* (defined as three or more episodes of unformed stools in 24 hours and *C. difficile* toxin positive stool), or colonoscopic or histopathologic findings of pseudomembranous colitis; *C. difficile* was the only clinical outcome tracked after patients’ ICU stay [18]. We also measured duration of mechanical ventilation; ICU and hospital stay; and ICU and hospital mortality.

### Antimicrobial administration

Antimicrobial therapy was recorded prospectively daily in each patient, and we calculated days of therapy as well as antimicrobial-free days in ICU.

### Follow-up

Patients were reviewed daily by the research coordinator in each ICU to collect baseline data (e.g., demographics, illness severity, advanced life support), daily data (e.g., study intervention administration and reasons for not administering, relevant medications including antimicrobials and prokinetics, VAP prevention co-interventions, culture results, clinical diagnoses, length of stay, mortality), and methods center data (e.g., infection adjudication forms). Research coordinators submitted relevant clinical, radiologic, and microbiologic data from patients who had clinically suspected VAP to the methods center. All other ICU-acquired infections were also documented and source data were collected. Any *Lactobacillus* species identified in a sterile site or cultured as the sole or predominant organism in a non-sterile site prompted discontinuation of the study product. Any reasons for protocol non-compliance were recorded daily while in ICU, along with contamination by probiotic administered outside the study protocol.

### Serious adverse events

A serious adverse event is defined as any adverse occurrence or event, or response to a drug or intervention, whether expected or not; that requires in-patient hospitalization or prolongation of existing hospitalization; that results in persistent or significant disability or incapacity; or is a congenital anomaly or birth defect; that is life threatening or results in death [19]. Serious adverse events were reported and documented in accordance with our guidelines for academic critical care trials of common interventions [20].

### Substudy no. 1: PROSPECT-TIMES

We conducted a multicenter time-in-motion study (PROSPECT-TIMES, Probiotics to prevent Severe Pneumonia and Endotracheal Colonization Trial—Time-In-Motion Evaluation Study) to document and describe the time and activities of the Research Coordinators who followed their first 10 randomized patients, concurrently documenting trial-related activities in 14 domains using a self-administered daily time management log.

### Substudy no. 2: PROSPECT capsule quality

We evaluated the content of the probiotic administered, taking one capsule from every 10 sheets (10 capsules per sheet) and culturing it in the Surette Laboratory at McMaster University. The study product was first rolled over a brain heart infusion agar medium (BD Difco, Franklin lakes, USA) to determine the presence of microorganisms on the exterior of the capsule follow by enumeration by serial dilution of the content of the capsule on de Man, Rogosa and Sharpe (BD Difco, Franklin lakes, USA) [21] and brain heart infusion agar media at the appropriate dilution. Subsequently, the numbers of colony-forming units were counted to determine the total number of colony-forming units per capsule and to evaluate whether organisms other than *Lactobacillus* were recovered.

### Substudy no. 3: serum markers of inflammation and microbiome feasibility study

From mechanically ventilated patients enrolled in PROSPECT in three centers in Hamilton, ON, local research coordinators obtained specimens on the day of enrollment, then each Monday, Wednesday and Friday during the patient’s ICU stay for the first 30 days, then Tuesday and Thursday thereafter for a maximum of 60 days. Specimens were procured, packaged in biohazard-safe material, and couriered via the interhospital within-city specimen transport system to the Surette and Bowdish Laboratories at McMaster University. We analyzed serum endotoxin activity assay and cytokine levels in blood, and microorganisms identified by culture-independent techniques in endotracheal aspirates, bronchial washings, gastric aspirates, and stool.

### Analysis

Baseline characteristics were presented by Group A versus Group B to evaluate the success of the randomization and reported as count (percentage) for categorical variables and mean (standard deviation) or median (first quartile to third quartile) for continuous variables depending on the distribution. Analyses of feasibility outcomes were made on the full cohort of 150 patients rather than according to the two randomized groups to retain the blinding; thus, no comparison between groups was needed to analyze feasibility outcomes. The feasibility outcomes were analyzed using descriptive statistics as follows: (1) mean (standard deviation) number recruited across all recruiting centers per month; (2) overall proportion of doses received divided by total doses prescribed; (3) total number of patients who ever received open-label probiotics as a proportion of all randomized patients; and (4) the proportion of patients who developed VAP according to the Centers for Disease Control and Prevention definition [13] in a subgroup of 99 patients as assessed by five adjudicators, with disagreements settled by consensus.

No interim analyses or subgroup analyses were planned, owing to the short duration and sample size of this pilot trial. All analysis was performed using SAS version 9.2.

### Sample size estimation

Our approach to sample size for the PROSPECT pilot trial was focused on feasibility. Thus, the sample size was based on interpreting the lower bound of confidence intervals around estimates [22], which in this case was around the feasibility objective of adherence to protocol, whereby successful adherence is defined as ≥80 % of prescribed intervention being administered. Using each patient as the unit of analysis, rather than doses prescribed, the PROSPECT pilot trial sample size calculation would have claimed feasibility if at least 80 % of the patients achieved successful protocol adherence. Assuming a preliminary estimate of 0.85, and a margin of error of 0.05, recruiting 150 patients will give a 95 % confidence interval estimate of the proportion of patients with successful protocol adherence with a lower bound of 0.80 [12].

### Management

To ensure protocol adherence and data quality, training sessions were held for research personnel at all sites using procedure manuals, standard operating procedures, slide presentations, and a study website. The PROSPECT steering committee was responsible for the conduct of this pilot trial, for upholding or modifying study procedures as needed, addressing challenges with protocol implementation, refining the protocol as needed, and reviewing the data.

The PROSPECT pilot trial was managed by McMaster University’s CLARITY research group, and was conducted under the auspices of the Canadian Critical Care Trials group [23, 24].

## Results

### Setting

The PROSPECT pilot trial was conducted between October 2013 and August 2014 in 14 ICUs: eight in Ontario [St. Joseph Healthcare, Hamilton; Hamilton Health Sciences, Hamilton (two ICUs), St. Michael’s Hospital, Toronto; Mount Sinai Hospital; Ottawa Health Research Institute (two ICUs); and the University Health Network—Toronto General Hospital]; one in Quebec [CHU de Québec-Université Laval; Hôpital de l’Enfant-Jésus site]; three in British Columbia [St Paul’s Hospital; Vancouver General; and the Royal Jubilee Hospital, Vancouver Island]; and two in the USA (Mayo Clinic, Rochester, MN and the St. John’s Mercy Hospital, St. Louis, MO).

### Enrollment

We enrolled 150 patients. The most common reason for non-enrollment of eligible patients was lack of availability of a substitute decision maker (65 situations, 51.6 %; Table 1). The consent rate for substitute decision makers or patients who were approached was 150/180 (83.3 %).

**Table 1:** Reasons for non-enrollment of 126 eligible patients

	Eligible non-enrolled patients
	*N* = 126
No substitute decision maker available	65 (51.6)
Patient or substitute decision maker declined	30 (23.8)
Missed	13 (10.3)
Coenrollment not pursued	7 (5.6)
Coenrollment prohibited	3 (2.4)
Language barrier	3 (2.4)
Physician decline (family stress)	2 (1.6)
Research coordinator did not approach (family stress)	1 (0.8)
Industry prohibited coenrollment	1 (0.8)
International visitor	1 (0.8)
Unknown reason	0 (0.0)

Enrolled patients were 60.0 (16.3) years of age [mean (standard deviation)], had an APACHE II score of 21.8 (7.9), and 62 (41.3 %) were female (Table 2). Patients had mostly medical (126, 84.0 %), but also surgical (8, 5.3 %), or trauma (16, 10.7 %) admitting diagnoses. At baseline, 84 (56.0 %) patients were receiving inotropes or vasopressors and 18 (12.0 %) were receiving dialysis. Baseline characteristics were comparable between Group A (*N* = 78 patients) and Group B (*N* = 72 patients).

**Table 2:** Baseline characteristics of randomized patients (150 patients, divided into Groups A and B, to maintain blinding)

	Group A	Group B	Total
	*N* = 78	*N* = 72	*N* = 150
Age (years), mean (standard deviation)	58.8 (17.0)	61.2 (15.4)	60.0 (16.3)
APACHE II, mean (standard deviation)	21.0 (8.4)	22.7 (7.3)	21.8 (7.9)
Female, *n* (%)	31 (39.7)	31 (43.1)	62 (41.3)
Type of patient, *n* (%)			
Medical	66 (84.6)	60 (83.3)	126 (84.0)
Surgical	3 (3.8)	5 (6.9)	8 (5.3)
Trauma	9 (11.5)	7 (9.7)	16 (10.7)
Admitting diagnosis, *n* (%)			
Cardiovascular	4 (5.1)	4 (5.6)	8 (5.3)
Respiratory	35 (44.9)	26 (36.1)	61 (40.7)
Gastrointestinal	2 (2.6)	0 (0.0)	2 (1.3)
Neurologic	8 (10.3)	10 (13.9)	18 (12.0)
Sepsis	16 (20.5)	17 (23.6)	33 (22.0)
Trauma	7 (9.0)	7 (9.7)	14 (9.3)
Metabolic	2 (2.6)	1 (1.4)	3 (2.0)
Hematologic	1 (1.3)	0 (0.0)	1 (0.7)
Renal	1 (1.3)	2 (2.8)	3 (2.0)
Other medical	2 (2.6)	4 (5.6)	6 (4.0)
Other surgical	0 (0.0)	1 (1.4)	1 (0.7)

### Feasibility outcomes

The results of the four feasibility outcomes were as follows: (1) the recruitment rate was 1.9 patients/center/month; (2) of 2107 patient-days in the ICU, a missed dose occurred in only 54 (2.6 %) of cases. Therefore, 97.4 % of the prescribed study product was administered as scheduled; (3) no enrolled patient received open-label probiotic after randomization; and (4) the adjudicated VAP rate was 19.2 %.

### Clinical outcomes

Table 3 summarizes all non-VAP infections that occurred in these patients after randomization. Bloodstream infections were the most common infection (19.3 %), followed by urinary tract infections (12.7 %) and skin and soft tissue infections (4.0 %).

**Table 3:** Secondary clinical outcomes in 150 patients after randomization

Source, *n* (%)	Following randomization: total *N* = 150
Blood culture positive	29 (19.3)
Intra-abdominal infection	5 (3.3)
Urinary culture positive	19 (12.7)
Skin or soft tissue infection	6 (4.0)
*Lactobacillus* species cultured from sterile site	1 (0.7)
Diarrhea (≥3 stools/day)	110 (73.3)
Antibiotic-associated diarrhea	95 (63.3)
*Clostridium difficile* infection	2 (1.3)

Patients had 1 (0–3) [median (interquartile range)] stools/day. Diarrhea defined as Bristol type 6 or 7 stools occurred in 133 (88.7 %) of patients, reflecting 56.6 % patient-days in ICU. Diarrhea defined as ≥3 stools/day occurred in 110 (73.3 %) patients, reflecting 555 (26.5 %) of patient-days. Antibiotic-associated diarrhea occurred in 95 (63.3 %) patients using the ≥3 stools/day definition. Overall, 56 (37.3 %) of patients used a fecal management device (rectal bag or tube) during their ICU stay, on average 4.5 days after admission. A fecal management device was used for 465 (22.1 %) of 2101 patient-days in ICU (missing stools data for 6 days).

*Clostridium difficile* infection occurred in two patients before ICU admission and before trial enrollment. Thereafter, two (1.3 %) patients developed this infection in the ICU and five (3.3 %) patients developed it after ICU discharge.

### Duration of mechanical ventilation, ICU and hospital stay, and mortality

The median (quartile 1 to quartile 3) duration of mechanical ventilation was 7 (3–14) days. The median duration of stay in ICU was 12 (7–18) days, and in hospital was 26 (14–44) days. There were 23/150 (15.3 %) ICU and 39/150 (26.0 %) hospital deaths. No patients were lost to follow-up.

### Antimicrobial administration

Antibiotic therapy was administered to 139 (92.7 %) patients, for 1333 (63.3 %) of 2107 patient-days in the ICU, for a duration of 7 (quartile 1 to quartile 3, 3–12) median days (Table 4). There were 660.7 days of antibiotic or antifungal therapy per 1000 ICU patient-days. There were 339.3 antimicrobial-free days per 1000 ICU patient-days.

**Table 4:** Antimicrobial administration for 150 patients over 2107 patient-days in the ICU

Daily receipt of antimicrobials, *n* (%)	*N* = 2107 patient-days in ICU
Antibiotic	1333 (63.3)
Antifungal	192 (9.1)
Antiviral	44 (2.1)
Antibiotic or antifungal	1392 (66.1)
Any antimicrobial (antibiotic or antifungal or antiviral)	1397 (66.3)
Antimicrobial (antibiotic or antifungal) free days	715 (33.9)
Ever in ICU up to day 60, *n* (%)	*N* = 150 patients
Antibiotic received	139 (92.7)
Antifungal received	14 (9.3)
Antiviral received	12 (8.0)
Antibiotic or antifungal received	139 (92.7)
Any antimicrobial (antibiotic or antifungal or antiviral) received	140 (93.3)
No antibiotic or antifungal ever received	11 (7.3)
Days of antimicrobial receipt, median (quartile 1 to quartile 3)	*N* = 139 patients
* Only in patients who received the antimicrobial*	
Days on antibiotic	7 (3–12)
Days on antifungal	10.5 (5–19)
Days on antiviral	2 (1.5–5)
Days on antibiotic or antifungal	8 (3–13)
Days on any antimicrobial (antibiotic or antifungal or antiviral)	7.5 (3–12.5)

### Serious adverse events

One immunocompetent patient had *Lactobacillus* species identified in a blood culture drawn from an arterial catheter after 4 days in the trial; the concurrent central venous catheter blood culture was negative. Although this patient remained asymptomatic, both catheters were removed and the patient was treated with ciprofloxacin for 10 days. As this was not classified as a serious adverse event [19], we did not unblind this patient’s allocation. It was unclear whether this represented an endogenous *L. rhamnosus* isolate that translocated from the gut, whether this was probiotic that translocated from the gut, or whether there had been contamination with probiotic to the hands of the person breaking the capsule then attending to the arterial catheter. This was classified as an adverse event [19, 25]. There were no serious adverse events in the PROSPECT pilot trial.

### Substudy no. 1: PROSPECT-TIMES

**Fig. 2:**
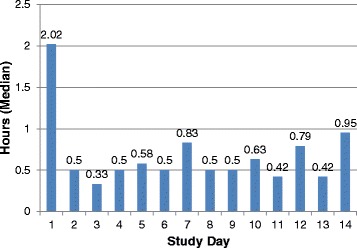
Average number of hours spent by the PROSPECT pilot trial research coordinator screening, obtaining consent, and enrolling patients per study day of the trial.

In seven participating centers, 62 patients were enrolled, taking an average of 9.2 hours of work per patient (range 3.2–28.5 hours/patient). Study day 1 activities (screening, consenting, and enrolling) took significantly longer than subsequent days in the trial (2.1 [standard deviation, 1.1] hours versus 0.76 [standard deviation, 0.71] hours, respectively [*P* < 0.01]) (Fig. 2) [26] and tended to spend fewer hours on each enrolled patient as the trial unfolded. On an average study day, research coordinators spent 0.9 (standard deviation, 0.8) hours per enrolled patient. Primary activities included data collection (55 %), ICU team communication (10 %), obtaining consent (6 %), responding to queries (6 %), and bedside charting (5 %).

### Substudy no. 2: PROSPECT capsule quality

**Fig. 3:**
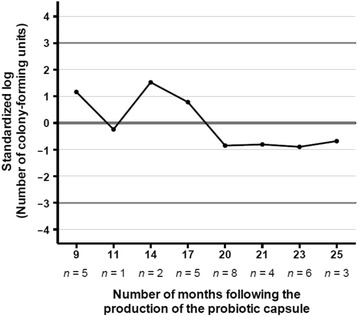
Capsule quality assurance results. The quality control protocol was performed on 34 probiotic capsules used in the PROSPECT pilot trial

We tested 34 probiotic capsules from 10 centers and used a process bar-chart to monitor the number of colony-forming units of *L. rhamnosus* GG (on a logarithmic scale) over 25 months. Each probiotic capsule contained at least 10^10^ colony-forming units of *L. rhamnosus* GG (10 on a logarithmic scale). The bacterial content of the capsules remained above the established threshold of 10^10^ colony-forming units and controlled within three standard deviations on the logarithmic scale for up to 25 months (Fig. 3).

### Substudy no. 3: Serum markers of inflammation and microbiome feasibility study

Overall, 938 samples were procured from 42 patients in three ICUs; 909 of these (96.9 %) were processed. Of the 29 specimens not processed, the reasons were: receipt beyond the 3 hour time frame for valid analysis (*n* = 10 specimens), no kits available (*n* = 7), no transportation due to holiday or inclement weather (*n* = 4), no laboratory staff due to conference (*n* = 4), lost in transport (*n* = 2), insufficient quantity (*n* = 1), and missed (*n* = 1).

We estimated on average that each specimen required 5 min to procure, package, and ship (shipping and processing times were not estimated). Also, for each day that any specimens were transported, we estimated 5 min to communicate with the laboratory and document specimens on the study log, and 10 min to make a return trip with the specimens from the ICU to the transportation office, for every two patients with specimens collected on that day. Sending specimens to the central laboratory took ICU research coordinators a mean of 58.2 (standard deviation, 31.7) min (total range, 20–180) per specimen collection day per site. For 42 patients, 122.2 total hours were spent on specimen procurement, packaging, and shipping.

## Discussion

We designed the PROSPECT pilot trial with four specific feasibility objectives in preparation for a future larger multicenter trial comparing probiotics with placebo. This pilot trial achieved prespecified feasibility objectives in four domains: (1) recruitment was 1.9 patients per month among actively recruiting centers—a total of 150 patients were randomized over 1 year, (2) protocol adherence was high—97 % of doses prescribed were actually received, (3) contamination with open-label probiotics did not occur, and (4) the VAP rate was 19 %. In this cohort, many patients had nosocomial infections, and most were prescribed antimicrobials and developed diarrhea. No enrolled patients were lost to follow-up.

We found that this pilot trial was a worthwhile investment and that its completion will serve to enhance research efficiency. This pilot trial will maximize the chance that the future main trial is designed rigorously, conducted safely and efficiently, and completed as planned [22, 27]. Other strengths of this study include concealed allocation, and blinding of all possible parties (patient, family, bedside clinicians, research personnel, and biostatistician) which helped to avoid unequal co-interventions, ascertainment bias, outcome modification, and analytic bias.

The three PROSPECT substudies were also informative. We documented that research staff spent an average of 1 hour per day on enrolled patients, which is useful for personnel and budgetary planning. Additional specimen collection for a mechanistic substudy on serum markers of inflammation and the microbiome took 1 hour per day. The quality assurance substudy yielded reassuring results in that the bacterial content of probiotic capsules tested remained above the protocol threshold of 10^10^ colony-forming units of *L. rhamnosus* GG up to 2 years after the manufacturing date; however, we plan to continue performing quality control microbiological analysis, evaluating one capsule from every tenth capsule sheet (1 %) during the main trial.

Limitations of this study include the modest sample size to address clinical outcomes, although enough patients were enrolled to address our feasibility objectives. Accordingly, no significance testing between groups was needed for analysis to evaluate the feasibility objectives, thereby retaining the blinding. The estimated 19 % incidence of VAP was based on a sample of two-thirds of enrolled patients, not the entire cohort, as our fourth feasibility objective was only to ensure that the rate was at least 10 %.

The PROSPECT pilot trial will be an internal pilot trial [27], and patients will be rolled into the main PROSPECT trial. Indeed, beyond the pilot trial, we launched the PROSPECT vanguard phase to avoid complete study cessation and a hiatus between the pilot trial completion and the main PROSPECT trial start-up. That is, after the PROSPECT pilot trial enrollment ended at 150 patients, we analyzed the pilot trial results, and continued to enrol 250 patients. We are now poised to launch the main PROSPECT trial.

The clinical and economic burden of VAP remains high [8] and the use of existing VAP prevention strategies is variable but disappointing in Canada [28]; thus, a simple, inexpensive, and safe prevention strategy could further reduce VAP rates. VAP continues to be a key ‘quality indicator’ for most hospitals in Canada (http://www.patientsafetyinstitute.ca/en/About/Programs/SHN/Pages/default.aspx), and is emphasized by the Institute for Healthcare Improvement (http://www.ihi.org/Pages/default.aspx), which motivates identification of inexpensive strategies to prevent VAP that can be successfully implemented and that do not involve antibiotics, given the worldwide resistance problem [29]. Recently, a Markov cost–benefit decision model determined that, from the hospital perspective, the VAP prevention strategy with the optimal cost–benefit ratio included probiotics, a suction endotracheal tube, and the revised Institute for Healthcare Improvement bundle, which excludes oral care [30].

## Conclusions

In summary, our results suggest that a large randomized controlled trial of probiotics in critically ill patients would be feasible. Probiotics remain a promising method to prevent VAP and other infections in critically ill patients, with biologic plausibility, clinical promise, and apparent cost-effectiveness, based on data available to date. Also, probiotics might have other salutary effects relevant to the antimicrobial stewardship movement, such as decreased initiation of antimicrobials and decreased duration of antimicrobial therapy. Probiotics constitute a promising method to prevent infection in critically ill patients. The large trial that is required to inform the overall effects of probiotics on clinical outcomes is now underway (NCT02462590).

## Abbreviations

CLARITY, Clinical Advances through Research and Information Translation; HIV, human immunodeficiency virus; ICU, intensive care unit; PROSPECT, Probiotics: Prevention of Severe Pneumonia and Endotracheal Colonization; VAP, ventilator-associated pneumonia

## Additional file

Additional file 1: PROSPECT pilot center research ethics board information. (DOCX 14 kb)
