# Supplementary material for: Probiotics: Prevention of Severe Pneumonia and Endotracheal Colonization Trial—PROSPECT: a pilot trial
Source: Trials. 2016 Aug 2;17:377. doi: 10.1186/s13063-016-1495-x (PMC4970233; doi:10.1186/s13063-016-1495-x)
Supplement: Additional file 1: — PROSPECT pilot center research ethics board information. (DOCX 14 kb) [file 13063_2016_1495_MOESM1_ESM.docx]

**Additional File 1:** PROSPECT Pilot Center Research Ethics Board (REB) Information

| **Center** | **Site Information** | **REB Information** |
| --- | --- | --- |
| 1 | St Joseph’s Healthcare Hamilton, Hamilton | Hamilton Integrated Research Ethics Board (HIREB) |
| 2 | Hamilton Health Science – Hamilton General Hospital, Hamilton | Hamilton Integrated Research Ethics Board (HIREB) |
| 3 | Hamilton Health Science – Jurvinski Hospital, Hamilton | Hamilton Integrated Research Ethics Board (HIREB) |
| 4 | St Michael’s Hospital, Toronto | St Michael’s Hospital Research Ethics Board |
| 5 | St. Paul’s Hospital, Vancouver | UBC Providence Health Care Research Ethics Board |
| 6 | Mount Sinai Hospital, Toronto | Mount Sinai Research Ethics Board |
| 7 | Centre Hospitalier Universitaire de Quebec – Hopital de L’Enfant-Jesus, Quebec City | Comite d'ethique de la recherché du centre hospitalier universitaire de Quebec |
| 8 | The Ottawa Hospital- Civic Campus, Ottawa | Ottawa Health Science Research Ethics Board |
| 9 | The Ottawa Hospital- General Campus, Ottawa | Ottawa Health Science Research Ethics Board |
| 10 | Vancouver General Hospital, Vancouver | Providence Health Care Research Ethics Board |
| 11 | University Health Network, Toronto Western Hospital, Toronto | University Health Network, Research Ethics Board |
| 12 | Mayo Clinic, Rochester, Minnesota | Mayo Clinic Institutional Review Board |
| 13 | Mercy Hospitals East Communities, St. Louis, Missouri | Mercy Hospital Saint Louis Institutional Review Board |
| 14 | Vancouver Island Health Authority, Victoria | Vancouver Island Health Authority Clinical Research Ethics Board |
